# Tannic acid reactivates HIV-1 latency by mediating CBX4 degradation

**DOI:** 10.1128/jvi.01173-24

**Published:** 2024-12-18

**Authors:** Cancan Chen, Zhihan Zhong, Wanying Zhang, Baijin Xia, Liyang Wu, Liting Liang, Yiwen Zhang, Hui Zhang, Xu Zhang, Ting Pan, Linghua Li, Bingfeng Liu

**Affiliations:** 1 Institute of Human Virology, Department of Pathogen Biology and Biosecurity, Key Laboratory of Tropical Disease Control of Ministry of Education, Guangdong Engineering Research Center for Antimicrobial Agent and Immunotechnology, Zhongshan School of Medicine, Sun Yat-sen University 74644 , Guangzhou, Guangdong, China; 2 Department of Pathology, The First Affiliated Hospital, Sun Yat-sen University, Guangzhou, Guangdong, China; 3 Infectious Diseases Center, Guangzhou Eighth People’s Hospital, Guangzhou Medical University 26468 , Guangzhou, Guangdong, China; 4 Qianyang Biomedical Research Institute, Guangzhou, Guangdong, China; 5 Shenzhen Key Laboratory for Systems Medicine in Inflammatory Diseases, School of Medicine, Shenzhen Campus of Sun Yat-sen University, Sun Yat-sen University 660329 , Shenzhen, Guangdong, China; The Ohio State University, Columbus, Ohio, USA

**Keywords:** tannic acid (TA), HIV-1 latent reservoir, reactivation, CBX4

## Abstract

HIV-1 can integrate viral DNA into host cell chromosomes and establish a long-term stable latent viral reservoir, a major obstacle in curing HIV-1 infection. The reactivation of latent proviruses with latency-reversing agents (LRAs) is a prerequisite for the eradication of viral reservoirs. Previous reports have shown that tannic acid (TA) exerts several biological functions, including antioxidant and antitumor activities. Here, we identified a novel function of TA as a reactivator of HIV-1 latency. TA showed similar features to the HIV-1 transactivator of transcription (Tat) and was able to reactivate a larger number of proviruses from various integration sites. TA also showed a strong synergistic effect with other LRAs acting on different signaling pathways. Further studies revealed that the polycomb repressive complex 1 component, chromobox protein homolog 4 (CBX4), is specifically degraded by TA through ubiquitination. CBX4 is associated with the tri-methylation at lysine 27 of histone H3 (H3K27me3) which was enriched on HIV-1 long terminal repeat regions. The TA-induced CBX4 degradation decreased the H3K27me3 enrichment and subsequently enhanced the transcriptional activity of the integrated proviruses. These results suggest that TA is an efficient LRA aiming to a new target for HIV-1 latency, which could be developed to eradicate latent proviruses.

**IMPORTANCE:**

HIV-1 remains a global health challenge, with its ability to integrate into the host genome and evade the effects of drugs. To overcome this obstacle, the “shock and kill” strategy was proposed, targeting the reactivation of latent HIV-1 for subsequent eradication through antiretroviral medication and immune system reinforcement. Here, we found a new reactivator for HIV-1 latency, tannic acid (TA), which can reactivate HIV-1 latency widely and deeply. Moreover, we demonstrated that TA could promote the interaction between the polycomb repressive complex 1 component CBX4 and the E3 ubiquitin ligase cullin 4A (CUL4A), resulting in CBX4 degradation through the ubiquitin-proteasome system. These events reduce H3K27me3 enrichment in the HIV-1 long terminal repeat region, thereby promoting HIV-1 transcription and ultimately reactivating HIV-1 latent infection. Our work may facilitate the identification of new latency-reversing agents and provide more theoretical evidence for the molecular mechanism of HIV-1 latency.

## INTRODUCTION

Currently, the major treatment strategy for person with HIV-1 (PWH) is suppressive combined antiretroviral therapy (ART). However, ART cannot completely eradicate the viral reservoir as HIV-1 can integrate into the host genomic DNA and establish HIV-1 latency (1–3). The mechanisms underlying HIV-1 latency have been extensively studied in recent decades (4, 5). Previous studies demonstrated that low levels of nuclear factor kappa-light-chain-enhancer of activated B cells (NF-κB) and nuclear factor of activated T-cells lead to HIV-1 latency (6, 7). Furthermore, recent studies have described other factors that can also affect HIV-1 latency, including epigenetic modifications, integration site distributions, and post-transcriptional regulation (8–10). Several studies have highlighted the determinant role of epigenetic modifications in HIV-1 latency. Both histone deacetylases (such as HDAC-1 and HDAC-2) and histone methyltransferases (such as G9a, Suv39H1, HP1 gamma, GLP, and EZH2) can facilitate HIV-1 provirus silencing (11–15).

The eradication of viral reservoirs is essential for curing HIV-1; however, because latently infected cells do not express viral antigens, their recognition and destruction by the immune system is difficult. The “shock and kill” strategy has been proposed as the major method by which the immune system eradicates viral reservoirs. The reactivation of latent viruses from the reservoir allows the immune surveillance system to recognize and eradicate HIV-1-expressing cells via cytotoxic T lymphocyte response or antibody-dependent cellular cytotoxicity (16–20). Effective activators of HIV-1 latency are crucial for this approach, and for a long time, the key focus of the strategy has been on how to effectively activate HIV-1 latent infection (21–25). Therefore, it is very meaningful to develop more safe and efficient latency-reversing agents (LRAs). Several LRAs have been developed based on these HIV-1 latency mechanisms, such as SAHA (Vorinostat), Panobinostat, Romidepsin, JQ-1, and broystatin-1. Some of these LRAs were obtained through high-throughput screening in a latently infected cell model. However, there are disadvantages to using LRAs, such as non-specific reactivation of the host cells, ineffectiveness in clinical trials, and other toxic side effects (26–31).

HIV-1 Tat is an early-phase viral transcription protein that specifically activates the HIV-1 promoter through interaction with transactivation response elements and the recruitment of important transcriptional factors, such as the P-TEFb complex, which contains CDK9 and cyclinT1 (32, 33). At the same time, Tat protein can promote HIV-1 transcriptional activation by reducing the level of H3K27me3 at the HIV-1 long terminal repeat (LTR) in a variety of ways, including by inducing phosphorylation of EZH2 or interacting with UTX-1 (34, 35). In a previous study, we developed a mutant Tat protein (R5M4) that can significantly attenuate cytotoxicity and immunogenicity while retaining potent transactivation and membrane-penetration activity. Combined with HDACi, R5M4 can activate highly genetically diverse and replication-competent proviruses from resting CD4^+^ T lymphocytes isolated from PWH receiving ART (36). However, some issues regarding druggability and pharmaceutical characteristics of R5M4 remain to be resolved. Therefore, we aimed to identify candidates from a patent drug library with equivalent activation potency and properties to the Tat protein. This study could contribute to the effectiveness and specificity of latency-reversing strategies in future clinical trials.

## RESULTS

### TA reactivates the latent HIV-1 reservoir from the J-Lat-Tat cell line and primary CD4^+^ T lymphocytes

**Fig 1:**
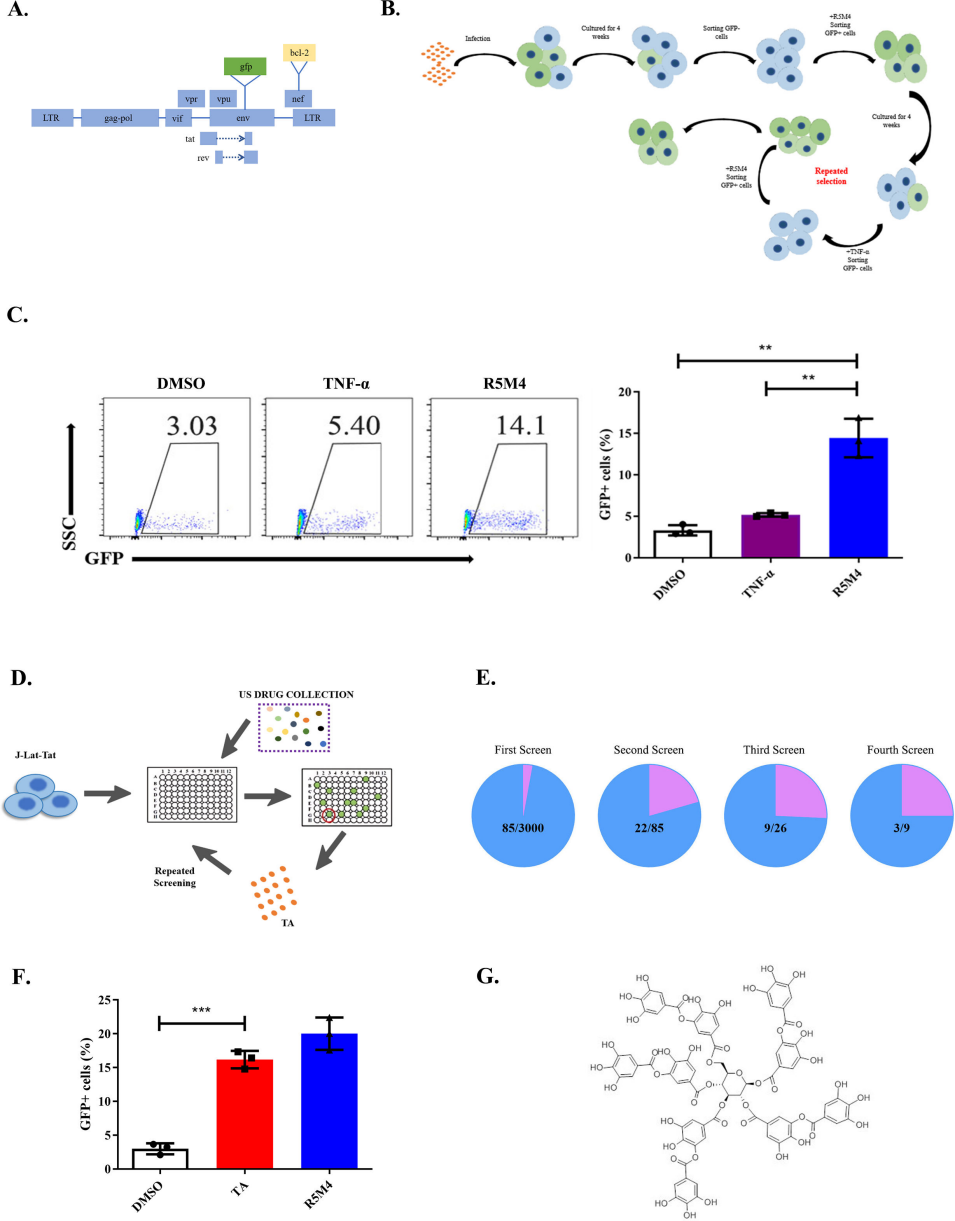
The processes of Tat-sensitive HIV-1 latent-infected cell line model (J-Lat-Tat) construction and Tat-like drugs screening. (**A**) The plasmid pNL4-3-ΔEnv-GFP-Bcl2 was used for packing HIV-1/VSV-G pseudoviruses. The bcl-2 gene was inserted into the nef region of pNL4-3-Env-GFP. (**B**) Strategy to construct Tat-sensitive HIV-1 latent-infected cell line model (J-Lat-Tat). (**C**) The reactivation efficiency of J-Lat-Tat cells with TNF-α and R5M4. The J-Lat-Tat cells were treated with 10 ng/mL TNF-α or 1 µmol/L R5M4 for 24 h, and then the reactivation efficiency was detected by flow cytometry. (**D and E**) High-throughput screening for Tat-like drugs. J-Lat-Tat treated with different drugs for 24 h, then tested the GFP-positive ratio with flow cytometry and selected effective drugs (**D**). The processes of screening were repeated four times and reduced the drug concentration gradually, from 200 to 10 µM. The number of candidate drugs is shown in (**E**). (**F**) TA reactivates J-Lat-Tat cells effectively. Ten micromolar tannic acid (TA) treated Tat-sensitive HIV-1 latently infected cells for 24 h, and the reactivation efficiency were tested by flow cytometry, and the results from three independent experiments were shown (mean ± SEM). (**G**) The molecular structure of TA.

J-Lat cell lines (J-Lat 6.3, 8.4, and 10.6), which are generated from single-cell clones and contain an integrated HIV-1 genome, are the most popular HIV-1 latently infected cell line models for LRA screening (37). However, these cell lines are highly sensitive to protein kinase C (PKC)-NF-κB pathway activators and, therefore, may be less sensitive to LRA candidates that exert their effects through other pathways. Our previously generated attenuated HIV-1 Tat protein, R5M4, is an effective LRA, as Tat can specifically regulate HIV-1 transcription. Therefore, we aimed to construct a latently infected cell line that is more sensitive to Tat than other LRAs. To achieve this, Jurkat cells were infected with the pseudotyped virus HIV-1_NL4-3_-ΔEnv-GFP-Bcl2, approximately 26% of green fluorescent protein (GFP)-positive cells were detected 4–5 days post-infection, and the GFP-positive cells were sorted and subsequently cultured. After a 2-week culture, approximately 84% of the GFP-positive Jurkat cells turned GFP negative. We sorted these GFP-negative populations, which were then stimulated with R5M4 and tumor necrosis factor alpha (TNF-α) successively, and obtained a group of cells that were more sensitive to R5M4 than to TNF-α. These cell populations were named J-Lat-Tat. Flow cytometry analysis showed that the reactivation efficiency of R5M4 in this cell line was about three times higher than that of TNF-α (Fig. 1A through C; Fig. S1). Here, we noticed that TNF-α is one of the most potent activators of HIV-1 latent infection. Consequently, even after sorting for TNF-α-negative responding cells, a small subset of the resulting population might still become activated upon re-stimulation with TNF-α.

This J-Lat-Tat cell model was adapted to identify candidates for HIV-1 latency reversal through treatment with a United States Food and Drug Administration (FDA)-approved drug library (US DRUGS COLLECTION) consisting of 1280 compounds. Following a 48 h treatment, the ratio of GFP-positive cells was determined by flow cytometry (Fig. 1D). In the first round of screening, we found 85 hit compounds at 1 mM, and their activating effects were validated at lower drug concentrations (200–10 µM) in three additional screening rounds. Tannic acid (TA) was identified in the fourth screening round, which effectively reactivated latent HIV-1-infected cells, showing >15% activation at 10 µM (Fig. 1E through G; Fig. S2A). TA is a complex polyphenolic compound found mainly in green tea, red wine, and coffee that has several effects, including antioxidant, antimicrobial, chelating, and anti-inflammatory properties (38–42). The concentration of tannins in red wines is estimated to range from 5 to 100 µM, depending on the variety. TA is one of the most important components of some traditional medicines and has been approved by the FDA as a safe food additive. Recent studies have shown, both *in vitro* and *in vivo*, that TA exerts antitumor functions by regulating mROS expression or promoting TRAIL-induced extrinsic apoptosis; it is also an inhibitor of CXCL12 (SDF-1alpha)/CXCR4 and decreases cancer stem cell formation (43–45).

**Fig 2:**
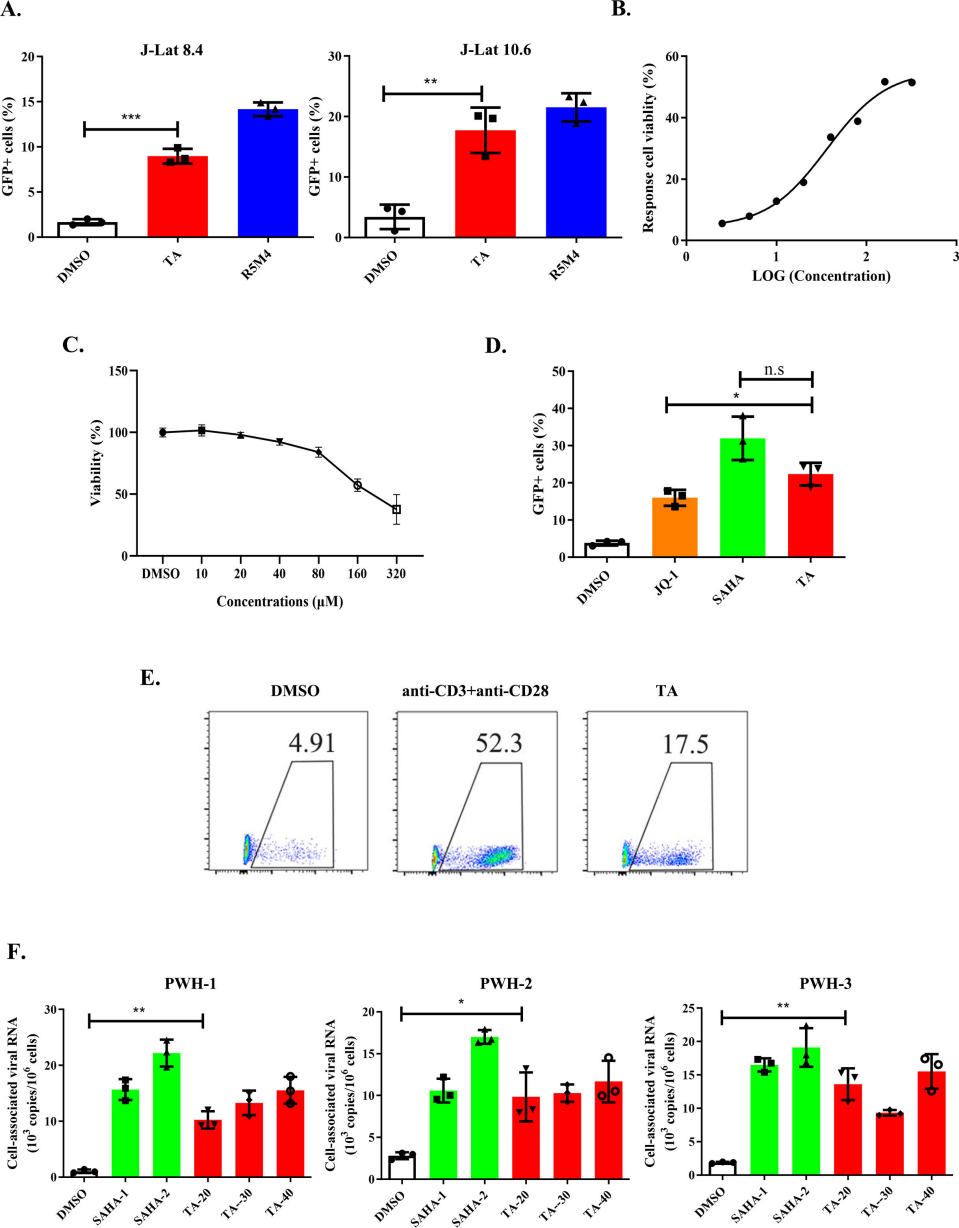
The reactivation effect of TA in different HIV-1 latency models. (**A**) The reactivation efficiency of TA in J-Lat 8.4 and J-Lat 10.6. J-Lat 8.4 and J-Lat 10.6 cells were treated with 10 µM TA for 24 h and detected the reactivation efficiency by flow cytometry, and the results from three independent experiments were shown (mean ± SEM). (**B**) The EC50 values of TA in J-Lat 10.6 cells. About 2.5–320 μM TA was added to J-Lat 10.6 cells for 24 h. (**C**) The cell viability of J-Lat 10.6 was measured using 3-(4,5-dimethylthiazol-2-yl)-5-(3-carboxymethoxyphenyl)-2-(4-sulfophenyl)-2H-tetrazolium (MTS) following TA treatment for 24 h at indicated concentrations. (**D**) The reactivation efficiency of TA, JQ-1, and SAHA in J-Lat 10.6 after 24 h of treatment at indicated concentrations. (**E**) HIV-1 latently infected primary cells were treated with 30 µM TA for 24 h and tested the GFP expression. (**F**) The reactivation effect of TA in the CD4^+^ T lymphocytes isolated from PWH. CD4^+^ T lymphocytes were isolated from three PWH and activated with 1 and 2 µM of SAHA or 20, 30, and 40 µM TA accordingly. After 72 h, the viral particles in cells were harvested, and viral RNA was analyzed by quantitative reverse transcription PCR (qRT-PCR).

To further confirm the reactivation effect on HIV-1 latency, we treated two different types of monoclonal cell lines (J-Lat 10.6 and J-Lat 8.4) with TA for 24 h. Both latently infected cell lines were significantly reactivated by TA**,** with a dose-dependent efficiency (Fig. 2A and B; Fig. S2B). To determine the potential cytotoxic effects of TA, the viability of J-Lat cells was assessed after treatment with increasing TA concentrations. The results showed that at the concentration range necessary for reactivating HIV-1 latency in J-Lat 10.6, TA exhibited low cell toxicity. A limited effect on cell viability was observed at concentrations of up to 80 µM (Fig. 2C).

SAHA and JQ-1 have been previously identified as LRAs and have already been investigated in several pre-clinical and clinical studies. We found that the TA activation ratio was not significantly different with SAHA but was more potent than that of JQ-1 at the indicated concentrations (Fig. 2D). To further examine whether TA could be effective in HIV-1 latently infected primary cells, we constructed an HIV-1 latency model using human primary CD4^+^ T lymphocytes, as previously described (36). We treated these cells with TA for 24 h and measured the ratio of GFP-positive cells, and the results showed that TA was also efficient in this primary cell model (Fig. 2E; Fig. S3). Moreover, we collected peripheral blood samples from PWH receiving ART for at least 2 years. Isolated resting CD4^+^ T cells were treated with TA or SAHA at the indicated concentrations for 72 h, and cell-associated viral RNA (CA-RNA) was tested. The results showed that the viral RNA level in the TA-treated group was significantly higher than that in the vehicle control group (Fig. 2F). Taken together, these data demonstrate that TA can effectively reactivate the HIV-1 latent reservoir, making it a potential LRA candidate.

### TA has a synergistic effect with other LRAs and broadly reactivates highly diverse latent proviruses

**Fig 3:**
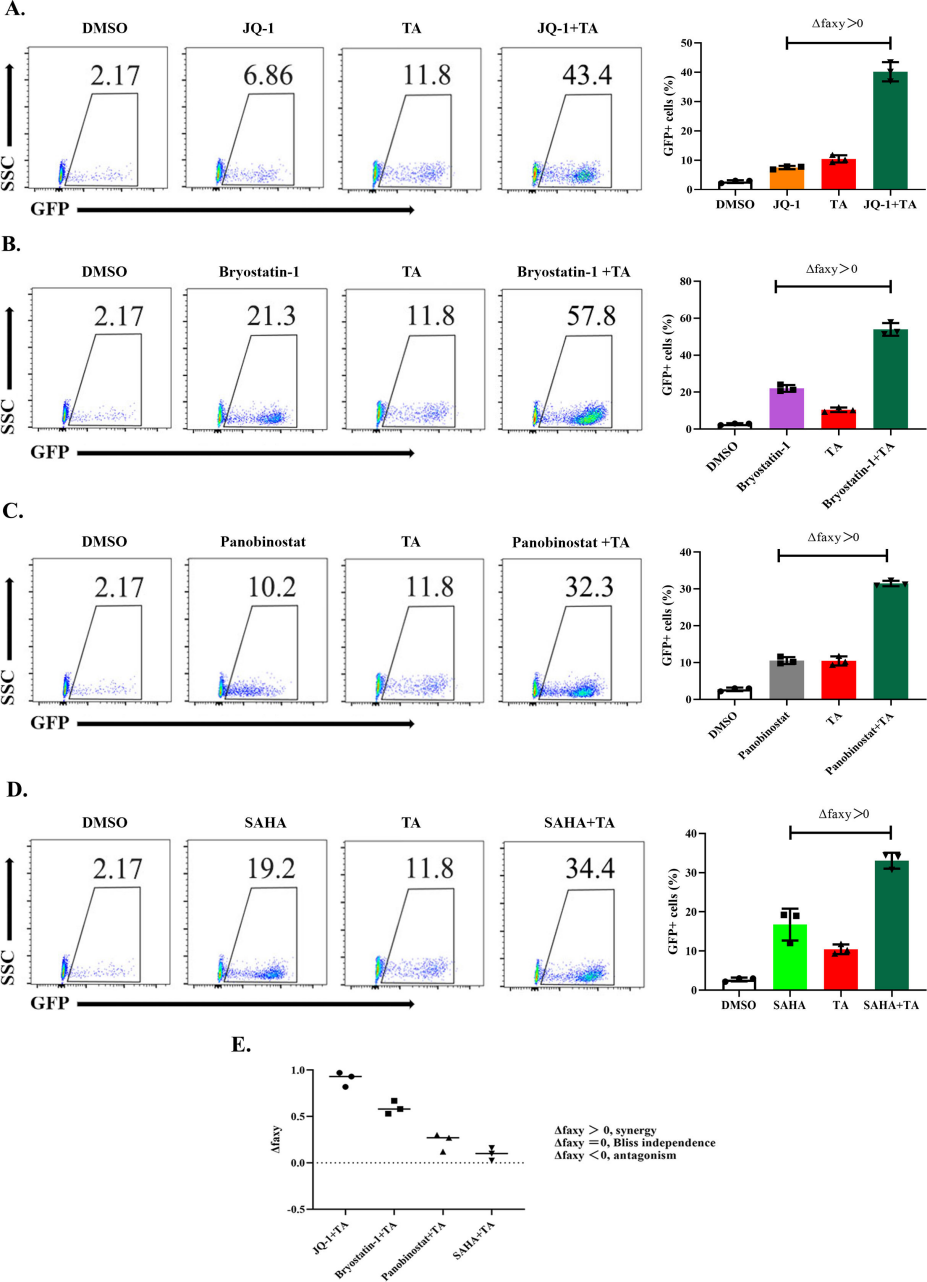
The synergistic effect of TA with other LRAs to reactivate latent HIV-1. (**A–D**) J-Lat 10.6 cells were treated with 10 µM TA or in combination with 0.5 µM JQ-1 (**A**), 0.5 µM Bryostatin-1 (**B**), 0.5 µM panobinostat (**C**), and 1 µM SAHA (**D**) for 24 h, and the percentage of GFP expressing cells was determined using flow cytometry. (**E**) TA synergizes with other LRAs to significantly increase HIV-1 expression in J-Lat 10.6 cells. Calculation of synergy for LRA combinations using the Bliss independence model. Dotted horizontal line signifies pure additive effect (Δfa_*xy*_ = 0). Synergy is defined as Δfa_*xy*_ > 0, while Δfa_*xy*_ < 0 indicates antagonism. Statistical significance was determined using a one-tailed ratio *t*-test comparing predicted and observed drug combination effects. The experiments presented in panels A–D were conducted concurrently, with each treatment independently repeated three times. The control groups for both DMSO and TA were identical.

Although several types of LRAs have been identified, including SAHA, JQ-1, and bryostatin-1, which function in different signaling pathways, maximizing the effectiveness with minimal cytotoxicity is nonetheless essential. Recent studies have shown that drug combinations could be a potential strategy to achieve high levels of latency reversal (46–49). Therefore, we evaluated the potential synergistic effect of TA with other LRAs that affect different signaling pathways and detect synergistic effects through the Bliss independence model (46). In this study, we combined TA with JQ-1, bryostatin-1, panobinostat, or SAHA to treat J-Lat 10.6 cells. Compared with single-drug treatment, combining TA with the four LRAs achieved significantly higher reactivation efficiency. The ratios of GFP-positive cells were at least twofold higher than that with single-drug treatment. In particular, when TA and JQ-1 were combined, the reactivation ratio was improved by nearly fivefold, we further confirmed the synergistic effect of TA in combination with these LRAs using the Bliss independence model (Fig. 3). The Bliss independence model is given by the formula: Δfa_*xy*_ = f_*xyO*_ − fa_*xyP*_, Δfa_xy_ > 0 indicates synergy, Δfa_*xy*_ = 0 suggests additive effect (Bliss independence), and Δfa_*xy*_ < 0 suggests antagonism. In samples from PWH, we also tested the combined effects of TA with different LRAs and found the same trend (Fig. S4).

**Fig 4:**
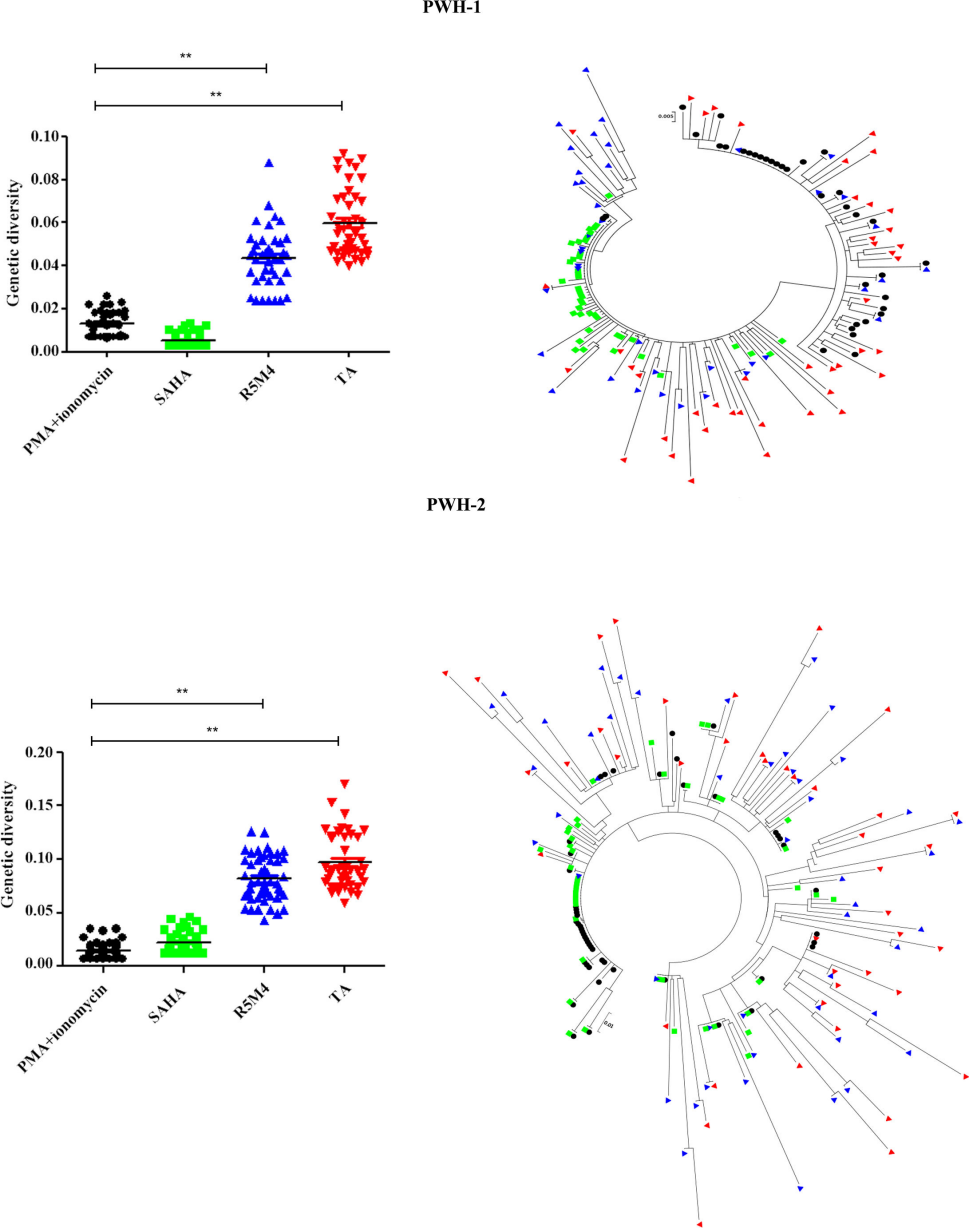
The genetic diversity of HIV-1 was induced by TA and other LRAs. HIV-1 strains were sampled from two virally activated subjects treated by PMA + ionomycin (black), SAHA (green), R5M4 (blue), and TA (red) respectively, and 50 clones were sequenced in each case. (Left) Each point represents the genetic distance between one given clone and the relevant entire population, and the horizontal bar indicates the mean. (Right) The bootstrap consensus trees were constructed based on HIV-1 sequences obtained from the corresponding PWH annotated in the left panel. The two-tailed Mann-Whitney *U*-test was used to compare the genetic diversities between different groups.

Previous studies have reported that attenuated Tat protein (R5M4) can stimulate several genetically diverse proviruses (36). As TA was screened from the Tat-sensitive latency model, we measured whether the phylogenetic tree of reactivated proviruses after TA treatment can be categorized into diverse lineages. The CD4^+^ T lymphocytes from PWH were treated with different LRAs, including PMA + ionomycin, SAHA, R5M4, or TA for 72 h. The analysis of the genetic diversity of CA-RNA showed that TA, which is similar to R5M4, can reactivate more HIV-1 quasispecies than SAHA and PMA + ionomycin (Fig. 4). These findings suggest that TA exhibits an excellent synergistic effect with other LRAs and can broadly activate a large number of genetically distinct HIV-1 proviruses.

### TA reactivates latent HIV-1 through mediation of CBX4 degradation to decrease the enrichment of H3K27me3 in LTR

**Fig 5:**
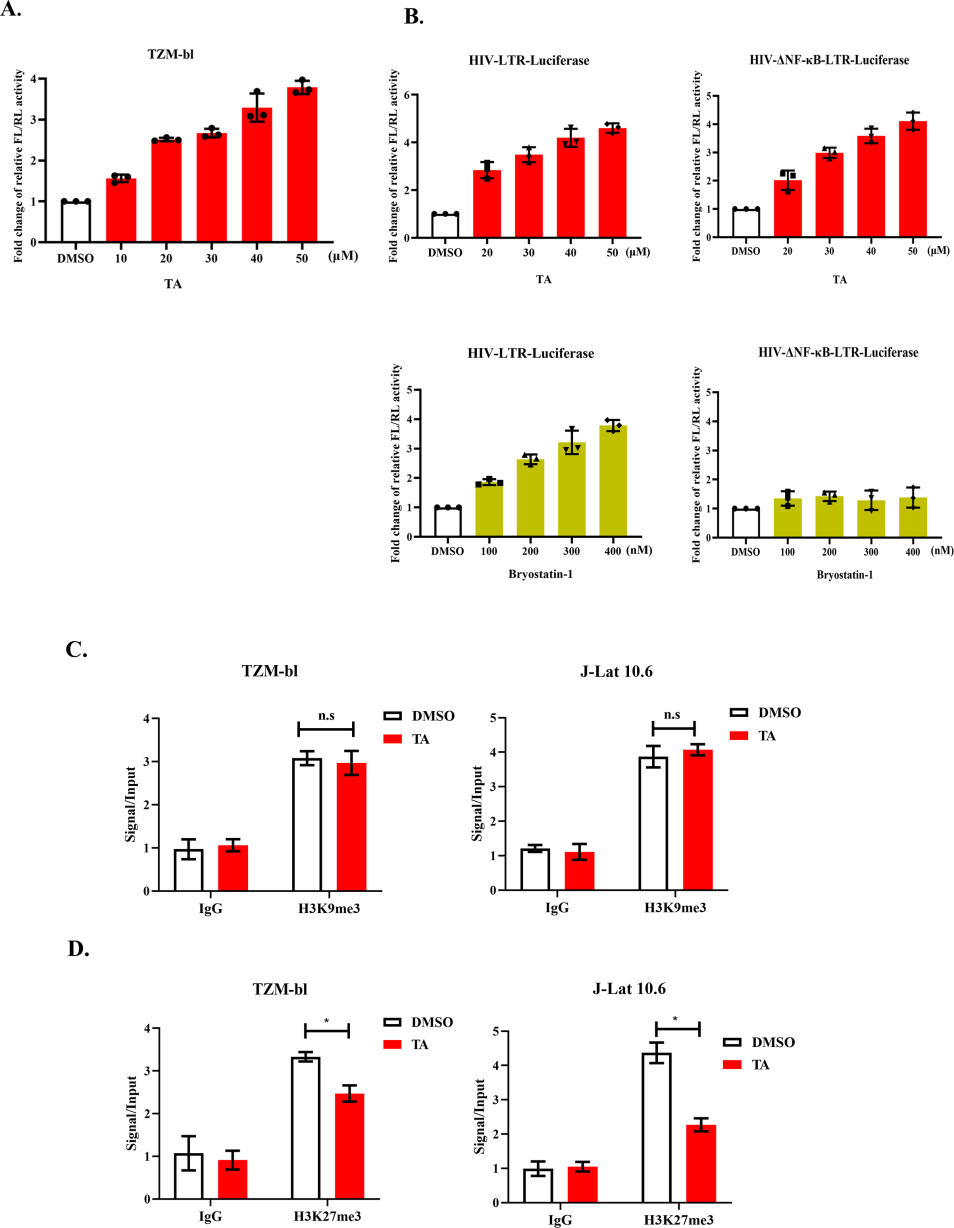
TA reactivates HIV-1 LTR transcription by decreasing the enrichment of H3K27me3. (**A**) pTK-Green Renilla Luc plasmids were transfected into TZM-bl cells, and after 6 h, the cells were treated with 0–50 μM TA, respectively, for 24 h, and dual-luciferase assay was used to detect the transcription of HIV-1 LTR. (**B**) TA activates HIV-1 LTR independent of NF-κB pathway. pcDNA3.1-Tat-HA, pTK-Green Renilla Luc, HIV-1-LTR-Luciferase, or HIV-1-ΔNF-κB-Luciferase were transfected into HeLa cells for 6 h. Then 0–50 μM TA or 0–400 nM Bryostatin-1 were treated with HeLa cells, respectively, for 24 h and tested the transcription of HIV-1 by using dual-luciferase assay. (**C and D**) The enrichment of H3K9me3 and H3K27me3 on HIV-1 LTR treated with TA. TZM-bl cells and J-Lat 10.6 cells were treated with TA or DMSO for 24 h, and chromatin immunoprecipitation (ChIP) assays with antibodies against H3K9me3 and H3K27me3 were performed both in TZM-bl cells and J-Lat 10.6 cells.

To determine whether TA can improve HIV-1 LTR reactivation, we treated TZM-bl cells with different concentrations of TA and evaluated the fold-change in Firefly Luciferase/Renilla Luciferase (FL/RL) activity by dual-fluorescence reporter assay, a positive correlation between the fold-change in FL/RL activity ratio and the efficacy of TA in activating HIV-1 LTR. The results of the dual-fluorescence reporter gene system confirmed that TA can activate LTR in a dose-dependent manner (Fig. 5A). To further test the pathways involved in TA reactivation of the latent reservoirs, we used the HIV-ΔNF-κB-LTR-luciferase system to detect the potential relation between TA and the NF-κB pathway. Compared to the positive control bryostatin-1, a reactivator dependent on the PKC-NF-κB pathway, we found that the NF-κB mutation had no significant influence on the ability of TA to reactivate HIV-1 LTR (Fig. 5B). This observation suggests that the TA reactivation of HIV-1 LTR is independent of the NF-κB pathway.

Previous studies showed that epigenetic modifications are responsible for HIV-1 latency, and several reports demonstrated that H3K9me3 and H3K27me3 modifications are important for LTR transcriptional activation (8, 14, 15). Therefore, we evaluated the enrichment of these epigenetic modifications with or without TA treatment. TZM-bl or J-Lat 10.6 cells were treated with TA or vehicle for 24 h, and the enrichment of H3K9me3 and H3K27me3 in HIV-1 LTR was determined by chromatin immunoprecipitation (ChIP) q-PCR. Results showed decreased enrichment of H3K27me3 in HIV-1 LTR in both TZM-bl and J-Lat 10.6 following TA treatment. However, TA treatment had no effect on H3K9me3 (Fig. 5C and D). These data suggest that TA can decrease the enrichment of H3K27me3 in HIV-1 LTR to promote transcription.

**Fig 6:**
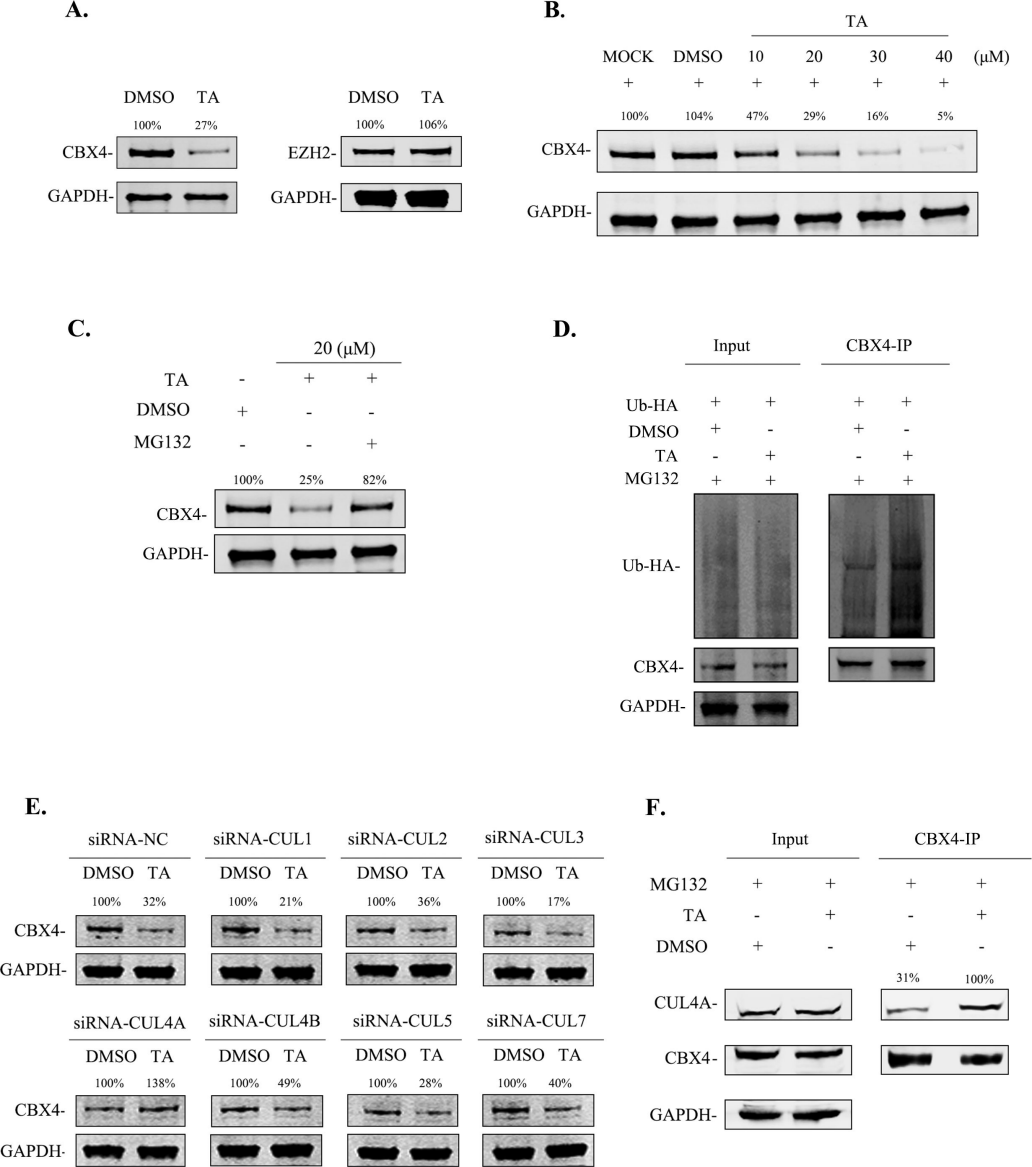
TA downregulates the expression of CBX4 via increasing the ubiquitination of CBX4. (**A**) Western blotting detected the expression of endogenous CBX4 and EZH2 proteins after TA treatment in J-Lat 10.6 cells. (**B**) J-Lat 10.6 cells were treated with 10–40 μM TA, and the expression of endogenous CBX4 was confirmed after 24 h treatment by western blotting with anti-CBX4 antibody. (**C**) J-Lat 10.6 cells were treated with 20 µM TA, with or without 4 µM MG132, for 16 h before cells collection, then detected the endogenous CBX4 expression with western blotting. (**D**) Four micrograms pcDNA3.1-Ub-HA was transfected into TZM-bl cells. After 6 h, cells were treated with 20 µM TA for 24 h, and 4 µM MG132 were added for 16 h before sample collection, analyzed by co-immunoprecipitation with anti-CBX4, and detected by western blotting with anti-CBX4 and anti-HA antibodies. (**E**) TZM-bl cells were transfected with 50 nM negative control siRNA or CUL1, CUL2, CUL3, CUL4A, CUL4B, CUL5, or CUL7-specific siRNAs, respectively. Then treated with 20 µM TA or DMSO, after 24 h, CBX4 expression was analyzed by western blotting assay. (**F**) J-Lat 10.6 cells were treated with 20 µM TA for 24 h, and 4 µM MG132 was added 16 h before cells collection. Samples were immunoprecipitated with anti-CBX4, then analyzed by western blotting with anti-CBX4 and anti-CUL4A antibodies.

Polycomb group (PcG) proteins are present in numerous organisms and comprise two core protein complexes, polycomb repressive complex 1 (PRC1) and polycomb repressive complex 2 (PRC2). These have been identified as important factors for the establishment and maintenance of post-translational histone modifications (50, 51). Core PRC1 members, namely, CBX2, CBX4, CBX6, CBX7, and CBX8, have two main biochemical functions: catalyzing H2A monoubiquitylation and trimethyl-H3K27 binding. These molecules have a similar conservative domain to bind with specific histone modification (52, 53). J-Lat cells were treated with TA for 24 h, and the levels of these proteins were evaluated by western blotting. We found a dose-dependent decrease in CBX4 levels after TA treatment; however, CBX2, CBX6, CBX7, and CBX8 levels remained unaltered after the treatment (Fig. 6A and B; Fig. S5). According to previous reports, CBX4 possesses SUMO E3 ligase activity and can SUMOylate several proteins such as C-terminal binding protein and *de novo* DNA methyltransferase 3a (54, 55). Additionally, our previous study identified that the CBX4 contributes to HIV-1 latency and recruits EZH2 to CBX4 bodies for SUMOylation, which enhances its H3K27 methyltransferase activity (56). These results demonstrate that CBX4 is a specific target of TA for the reactivation of HIV-1 latently infected cells.

### TA degrades CBX4 through CUL4A-mediated ubiquitination

To understand the underlying mechanism of TA-induced CBX4 degradation, we treated J-Lat 10.6 cells with dimethyl sulfoxide (DMSO) or TA and evaluated CBX4 mRNA levels. We found no significant differences in CBX4 expression after TA treatment, indicating that TA likely degraded CBX4 directly in the protein level (Fig. S6A). J-Lat cells were treated with TA, and MG132 was added to the cell culture for 16 h. The results showed that TA could effectively degrade CBX4; however, the protein levels recovered significantly after MG132 treatment (Fig. 6C). Furthermore, we observed that CBX4 ubiquitination was significantly increased after TA treatment, suggesting that the TA-induced CBX4 degradation occurs through the ubiquitin-proteasome system (UPS; Fig. 6D). Cullin-based RING ligases are the largest family of E3 ligases that are related to ubiquitination (57). We synthesized siRNA-CUL1, siRNA-CUL2, siRNA-CUL3, siRNA-CUL4A, siRNA-CUL4B, siRNA-CUL5, and siRNA-CUL7 and transfected them into TZM-bl cells and then treated with TA for 24 h. The results showed that CBX4 degradation was blocked when CUL4A was knocked down. The knockdown of other Cullin proteins had no effect on TA-induced CBX4 degradation (Fig. 6E). We further constructed a pEGFP-CBX4 expression plasmid and co-transfected siRNA-CUL3, siRNA-CUL4A, and siRNA-CUL4B into HeLa cells and subsequently treated them with TA and DMSO. We observed that the CBX4-GFP expression was significantly decreased by TA in si-NC and si-CUL3 groups; however, when endogenous CUL4A was knocked down, the expression of CBX4-GFP was not affected by TA (Fig. S6B). We hypothesized that TA mediates CBX4 ubiquitination by promoting CBX4 and CUL4A binding, and the coimmunoprecipitation (co-IP) results confirmed our assumption (Fig. 6F). Based on these results, we speculate that TA degrades CBX4 indirectly, thereby promoting CBX4 ubiquitination through the E3 ligase CUL4A and its consequent degradation by the UPS.

## DISCUSSION

Latent infection of HIV-1 in resting CD4^+^ T lymphocytes is the major obstacle in virus eradication in PWH receiving cART. The “shock and kill” approach has been extensively studied as a potential strategy to tackle this problem. Until now, the relevant research has never stopped, and the researchers are also trying to draw it closer from theory to clinical practice (4–6, 16, 23–25, 58, 59). Highly efficient LRAs are a prerequisite for this strategy. Although various latency reactivators have been identified, including SAHA, bryostatin-1, and JQ-1, they have uneven efficiency and act through different signaling pathways. However, every LRA has some disadvantages, such as low efficiency or the risk of inducing host gene overexpression (19, 27, 31). Therefore, identifying reactivators of HIV-1 latency that are more specific, stable, and safe is essential. The Tat protein is a key factor for HIV-1 transcriptional activation and one of the early expression proteins of HIV-1. The Tat can recruit related transcription factors by combining with the *cis*-element TAR of HIV-1, thus specifically re-activating and promoting HIV-1 mRNA transcription. In our previous study, we found that attenuated Tat protein R5M4 was highly effective in HIV-1 latency reactivation, which suggests the possibility of identifying additional Tat-like small-molecule agents (36, 60, 61).

In this study, we infected Jurkat cells with pseudotyped virus HIV-1NL4-3-ΔEnv-GFP-Bcl2 to obtain a latent HIV-1 infection model that is more sensitive to R5M4 than to TNF-α (TNF-α is used as a reactivator in the construction of several HIV-1 latent infection cell models). This cell model is sensitive to R5M4; however, it has a drawback in that its reactivation efficiency decreases during passaging. Using this model, we identified a novel Tat-like HIV-1 latency reactivator, TA, a naturally occurring, plant-derived polyphenol found in several herbaceous and woody plants. TA is safe and natural, and some reports have estimated that 100 mg of TA is the approximate amount found in one cup of tea (250 mL) (44, 62–64). According to our results, the EC50 of TA for latency reversal is approximately 34 µM; although this concentration of TA is relatively high, it is safe at the concentration for the organism.

In recent years, the combined use of HIV-1 reactivators has become a new trend in HIV-1 reactivation, as a single reactivator has limited efficiency. A suitable combination can effectively improve reactivation efficiency (46–49, 65, 66). Therefore, we evaluated the potential of TA in combination with other HIV-1 LRAs, including JQ-1, bryostatin-1, SAHA, and panobinostat. When combined with other reactivators, TA shows a synergistic effect, significantly increasing the reactivation efficiency at low concentrations. JQ-1, bryostatin-1, and panobinostat are different types of HIV-1 latency-reversing drugs and act through different mechanisms. These results indicate that TA can not only be used as an HIV-1 latency reactivation agent but also as a candidate adjuvant drug for other reactivators. This expands the application range of TA and increases its practical application. The potent effect of LRA combinations indicates that the co-administration of different LRAs could provide a suitable approach for reducing viral reservoirs.

Additionally, we confirmed that TA could activate a larger number of HIV-1 proviruses at different locations, similar to R5M4. This phenomenon means that TA has some functional similarities with R5M4 and also reflects a broader reactivation ability in HIV-1 latently infected cells, compared to some conventional reactivators. These results further demonstrate that TA functions as a Tat-like drug with good application prospects. Furthermore, we revealed that TA transcriptional activation of HIV-1 LTR is independent of the NF-κB pathway but mediated by a decrease in H3K27me3. H3K27me3 modification on the HIV-1 LTR is a key factor in the establishment and maintenance of HIV-1 latent infection (67). Typically, the LTRs of latent proviruses accumulate high levels of methylated histones, while the down-regulation of H3K27me3 levels in HIV-1 LTR is closely related to the activation of latent HIV-1 (15, 68, 69). Heterogeneous responses to TNF-α stimulation have been observed in HIV-1 latently infected cell lines, with those insensitive to TNF-α reactivation potentially linked to elevated H3K27me3 levels within the HIV-1 LTR region (15). Previous studies indicate that Tat reduces H3K27me3 levels at the HIV-1 LTR, and this reduction is enhanced with increasing doses of the Tat plasmid (34, 35). The impact of Tat expression against H3K27me3 of HIV-1 LTR is similar to that of TA. We speculated that this is one of the common properties of the Tat and TA reactivation mechanisms. Additionally, we found that the PRC1 core member, CBX4, is a specific TA target, as TA degraded its expression in a dose-dependent manner. Our previous research confirmed that CBX4 bridges PRC1 and PRC2, resulting in the synergistic maintenance of HIV-1 latency (56). In the present study, we found that TA can promote E3 ligase CUL4A-mediated ubiquitination of CBX4, targeting it for degradation by the proteasome. In mammals, CBX4 contains a highly conserved N-terminal chromodomain that can bind to H3K9me3 and H3K27me3, thus maintaining their suppressive epigenetic modifications on the HIV-1 LTR. Additionally, CBX4 recruits EZH2 and SUMO4 to CBX4 bodies, resulting in SUMOylation of EZH2, which in turn significantly enhances the methyltransferase activity of EZH2 on H3K27me3 (56, 70, 71). This means that in the latent state, PRC1 recruits H3K27me3 to the HIV-1 LTR to suppress HIV-1 transcription (72). When HIV-1 latently infected cells were treated with TA, the expression of CBX4 was inhibited via CUL4A-mediated ubiquitination. This might disrupt the structure and stability of PRC1 and lead to the decreased enrichment of H3K27me3 in HIV-1LTR, leading to the eventual reactivation of HIV-1.

TA has previously demonstrated the ability to prevent HIV entry into cells and to suppress the activity of reverse transcriptase (RT), indicating its potential to interfere with the early stages of viral infection (73, 74). Additionally, TA’s ability to reactivate latent HIV-1 by degrading CBX4 and reducing H3K27me3 enrichment introduces a new therapeutic avenue. The antiviral and latency-reactivation effects of TA seem to involve different mechanisms, which could be used in combination therapies. This strategy could involve using TA to reactivate latent reservoirs, followed by its antiviral action or pairing it with other antiretroviral drugs to target the reactivated virus. Precise timing and coordination of such interventions, possibly with immune-boosting agents or other “kill” strategies, could enhance the clearance of reactivated viruses.

**Fig 7:**
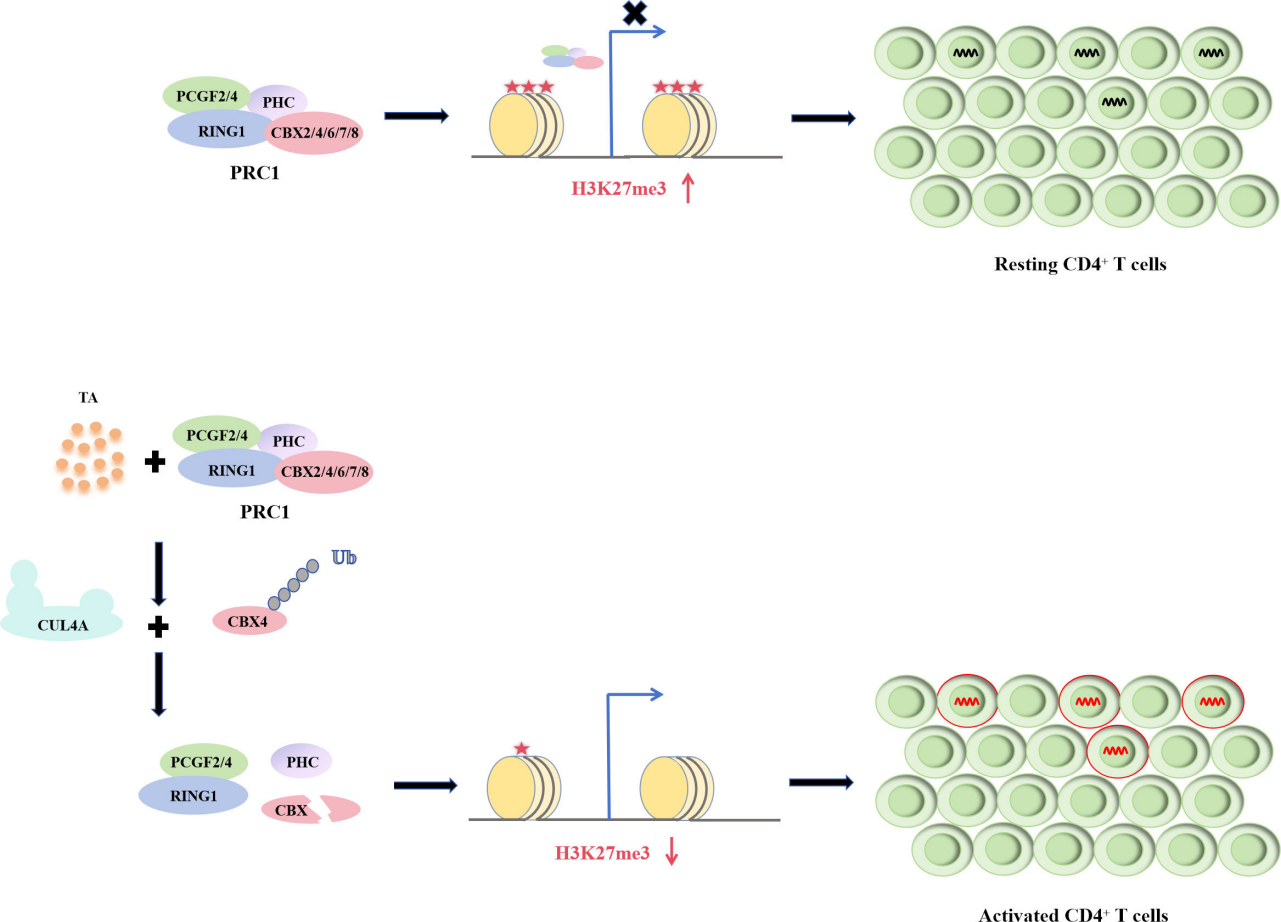
The potential mechanism of TA reactivates HIV-1 latency. In the absence of TA, the PRC1 complex is stable, which can promote the enrichment of H3K27me3 in HIV-1 LTR and contribute to the maintenance of HIV-1 latency. In the presence of TA, the binding between CUL4A and CBX4 is enhanced, which promotes ubiquitination and triggers the proteasomal degradation of CBX4. TA treatment can disturb the stability of PRC1 complex and decrease the enrichment of H3K27me3 in HIV-1 LTR. Consequently, HIV-1 latency is effectively reactivated.

In conclusion, this study introduces TA as a novel reactivator of HIV-1 latency, a critical step in the “shock and kill” strategy aimed at eradicating the virus. Our findings reveal that TA not only robustly reactivates latent HIV-1 but also targets the PRC1 complex by promoting the interaction between CBX4 and CUL4A, leading to CBX4 degradation and subsequent increase in HIV-1 transcription.(Fig. 7) This mechanism results in the reduction of H3K27me3 in the HIV-1 LTR region, a significant epigenetic change that aids in reactivating latent infections. The PRC-mediated HIV-1 latency has led us to develop novel LRAs. Currently, there are very few LRAs targeting PcG proteins. Notably, 3-deazaneplanocin A (DZNep), an EZH2 inhibitor, has been proven to effectively activate latent HIV-1 infection in CD4^+^ T cells and Jurkat T cells (75–78). TA not only enriches the variety of LRAs targeting PcG proteins but also offers significant potential for combination use in subsequent related studies. However, the *in vivo* translation of these findings and the long-term effects of TA on HIV-1 latency remain to be determined. Future research should focus on the optimization of TA as an LRA, exploring its combination with other epigenetic modifiers and assessing its safety and efficacy in preclinical models. This will provide a solid foundation for the potential clinical application of TA in HIV-1 eradication strategies.

## MATERIALS AND METHODS

### Drugs

In this study, the US Drug Collection, which was expanded to 1,280 drugs that have reached clinical trial stages in the USA, was used for the high throughput screening. Each compound has been assigned USAN or USP status and is included in the USP Dictionary (U.S. Pharmacopeia). In this experiment, different concentrations of TA were used to clarify the phenotype, and the concentrations of 10–30 μM were used for subsequent mechanistic experiments. SAHA (1 µM), JQ-1 (0.5 µM), Bryostatin-1 (0.5 µM), and panobinostat (0.5 µM) were used as controls for reactivation, and the concentrations were selected according to previous relevant studies (79–82).

### Plasmids and siRNA synthesis

The plasmids pNL4-3-ΔEnv-GFP-Bcl2, pHIV-ΔNF-κB-Pro-Luc, pcDNA3.1-Tat-HA, and pcDNA3.1-Ub-HA were previously constructed by our lab (36, 83, 84). CBX4-encoding cDNA with 3x-Flag tagging on the C-terminus was generated by reverse transcription-PCR (RT-PCR) with the mRNA of HEK293T cells as the template. Then the tagged CBX4 was inserted into pcDNA3.1 vector. CBX4 was amplified via PCR from pcDNA3.1-CBX4-3FLAG plasmid and inserted into pEGFP-C1 vector to generate pEGFP-CBX4-C1. The small interfering RNAs (siRNAs) targeting cullin 1 (CUL1), cullin 2 (CUL2), cullin 3 (CUL3), cullin 4A (CUL4A), cullin 4B (CUL4B), cullin 5 (CUL5), cullin 7 (CUL7), and non-related scramble sequence as negative control were designed and synthesized by Ribobio (Guangzhou, China).

### Cells and transfection

TZM-bl cells were obtained from AIDS Reference Reagent Program, NIH, which harbors an HIV-1 promoter-driven luciferase gene. HeLa cells and HEK293T cells were obtained from the American Type Culture Collection (ATCC). They were maintained in Dulbecco’s modified Eagle medium (DMEM; Hyclone) supplemented with 10% fetal bovine serum (FBS; Invitrogen) and 1% penicillin-streptomycin. These cells were transfected with the indicated plasmids or siRNAs by lipofectamine 2000 (Invitrogen). The procedures described by the manufacturer were followed. J-Lat 6.3, 8.4, and 10.6 cells were obtained from Dr. Robert F. Siliciano (Department of Medicine, Johns Hopkins University School of Medicine, Baltimore, MD, USA) Laboratory. Jurkat cells were purchased from cell bank of the Chinese Academy of Science, Shanghai. Human primary CD4^+^ T cells, J-Lat cells, and Jurkat cells were cultured in RPMI 1640 supplemented with 1% penicillin-streptomycin and 10% FBS. All cells were grown at 37°C with 5% CO_2_ and had been tested for mycoplasma using a PCR assay and were mycoplasma-free.

### Construction of Tat-sensitive HIV-1 latently infected cells (J-Lat-Tat)

The pNL4-3-ΔEnv-GFP-Bcl2 and pMD2.G were co-transfected into HEK293T cells to generate HIV-1/VSV-G pseudoviruses. Jurkat cells were infected with pseudoviruses for 4–5 days, and the infection efficiency was determined to be greater than 25% based on GFP expression. Then, the cells were cultured for 4 weeks under normal conditions until the GFP expression ratio was less than 5% and sorted the GFP-negative cells, which were then expanded in RPMI-1640 culture medium. The cells were stimulated with 1 µmol/mL R5M4 protein, and the GFP-positive cells were sorted and cultured for 4 weeks. The cells were treated with 10 ng/mL TNF-α, and the GFP-negative cells were sorted out. Subsequently, the procedures of R5M4 and TNF-α stimulation, followed by GFP-negative sorting, were repeated twice to obtain Tat-sensitive HIV-1 latently infected cells (J-Lat-Tat).

### MTS [3-(4,5-dimethylthiazol-2-yl)-5-(3-carboxymethoxyphenyl)-2-(4-sulfophenyl)-2H-tetrazolium] assay

The J-Lat 10.6 cells were cultured at a density of 5 × 10^4^ cells per well in 96-well cell culture plates and treated with TA in different concentrations. After 2 days, Cell Titer 96 AQueous One Solution (Promega, Madison, WI) was added to each well according to the instructions of the manufacturer. After treatment at 37°C with 5% CO_2_ for 2 h, the cell viability was tested by measuring the absorbance at 490 nm.

### Purification of human primary CD4^+^ T lymphocytes

The peripheral blood mononuclear cells (PBMCs) were isolated from healthy human donors by Ficoll gradient centrifugation. Human primary CD4^+^ T lymphocytes were then purified with a human CD4^+^ T-cell isolation kit (BD Biosciences). The isolated human primary CD4^+^ T lymphocytes were cultured in the conditioned RPMI 1640 medium (Hyclone) and stimulated with interleukin-2 (IL-2, R&D Systems), 1 µg/mL anti-CD3 antibody (BD Biosciences), and 2 µg/mL anti-CD28 antibody (BD Biosciences) for 48 h. Then, the cells were washed three times with PBS buffer and cultured in the presence of IL-2 (10 ng/mL).

### Construction of latently infected model from primary CD4^+^ T lymphocytes

The pNL4-3-ΔEnv-GFP-Bcl2 and pMD2.G were co-transfected into HEK293T cells to generate HIV-1/VSV-G pseudoviruses. In parallel, the primary CD4^+^ T lymphocytes were isolated from PBMCs of healthy donors and activated with 1 µg/mL anti-CD3 and 2 µg/mL anti-CD28 antibodies. These cells were then infected with the pseudoviruses. After 4 days, GFP-positive cells were sorted out. We then added anti-CD3, anti-CD28, and 10 ng/mL IL-2 and cultured the cells for 1 week for expansion. In the following 2–3 weeks, we gradually reduced the concentration and frequency of IL-2 supplementation, and the cells were maintained without IL-2 for 1 more week. The GFP-negative cells with more than 99.9% purity were isolated using fluorescence-activated cell sorting and subjected to activation by various reagents.

### Quantitative real-time RT-PCR analysis

The HIV-1-infected individuals with the blood plasma viral RNA less than 20 copies/mL and a number of CD4^+^ T lymphocytes higher than 200 cells/µL were recruited. The PBMCs were isolated through Ficoll gradient centrifugation, and CD4^+^ T lymphocytes were then purified with a human CD4^+^ T-cell isolation kit according to the manufacturer’s instructions (BD Biosciences). The isolated CD4^+^ T lymphocytes were then maintained in the conditioned RPMI 1640 medium (Hyclone) and treated with TA or SAHA. Cells were harvested after 3 days and detected the viral copies by real-time quantitative RT-PCR (qRT-PCR). RNA was isolated with Trizol reagent (Life Technologies) and then subjected to cDNA synthesis using PrimeScript RT reagent Kit (Takara). All primers were annealed at 37°C, and RT was processed at 42°C. Quantitative PCR was performed with SYBR Premix ExTaq II Kit (Takara) by following the manufacturer’s instructions. The expression of HIV-1 unspliced RNAs was determined by real-time qRT-PCR with the primer pair SK38 (5′-ATAATCCACCTATCCCAGTAGGAGAAA-3′) and SK39 (5′-TTTGGTCCTTGTCTTATGTCCAGAATGC-3′). An *in vitro*-synthesized HIV-1 RNA, after quantification, was used as the external control for measuring CA-RNA (85). Quantification was normalized to the housekeeping gene GAPDH.

### co-IP and western blotting

The 1.5 × 10^6^ TZM-bl cells were plated onto 60 mm cell culture dish. Twenty-four hours later, the cells used for ubiquitination studies were transfected with 4 µg of pcDNA3.1-Ub-HA. After 6 h, the cells were treated with TA or DMSO. Sixteen hours before we collected the cells, we added MG132 into the TZM-bl cells. The cells used for studying the interaction between CBX4 and CUL4A are treated the same way without transfection. At the appropriate time points, cells were collected and disrupted with radioimmunoprecipitation assay (RIPA) lysis buffer (150 mM NaCl, 50 mM Tris-HCl [pH 7.5], 1 mM EDTA, 1% NP40, and 0.5% Triton X-100) containing protease inhibitor cocktail (PIC; Sigma) and RNaseOut (Invitrogen) for 30 min on ice. The cell lysates were clarified with centrifugation at 12,000 g for 10 min at 4°C. BeyoMag Protein A + G magnetic beads (Beyotime, P2108-5mL) and anti-CBX4 antibody (Abcam, ab242149) were incubated at 4°C for 2 h. The supernatants were pre-cleared with agarose beads and then mixed with beads that had been incubated with anti-CBX4 antibody and incubated at 4°C for 4 h to overnight. The beads were washed five times with cold lysis buffer at 4°C, followed by washing four times with cold lysis buffer and eluting in gel loading buffer. As indicated, the beads were treated with RNase mixture (DNase-free, Roche; 20 µg/mL) and incubated at 37°C for 30 min. The immunoprecipitated samples were analyzed by SDS-PAGE and detected by western blotting with anti-CBX4 antibody (rabbit monoclonal antibody, Abcam, ab242149), anti-GAPDH antibody (rabbit polyclonal, Proteintech Group, 10494–1-AP), anti-HA (rabbit polyclonal, Proteintech Group, 51064–2-AP), or anti-CUL4A antibody (Rabbit monoclonal antibody, SAB, 49742). Images were analyzed by using Quantity One Software (Bio-Rad). Quantity One program (Bio-rad) was used to quantify the western blotting results.

### Dual-luciferase assay

Twenty-four hours before transfection, TZM-bl cells (2.5 × 10^4^ cells per well) or HeLa cells (2.5 × 10^4^ cells per well) were seeded into 48-well plates. To perform the reporter assay for wild type or mutated HIV-1 promoter activities, 100 ng of pTK-Green Renilla Luc and 2 ng of pcDNA3.1-Tat-HA were co-transfected into TZM-bl cells. After 6 h, the cells were treated with TA at different concentrations. One hundred nanograms of pTK-Green Renilla Luc, 2 ng of pcDNA3.1-Tat-HA, and 500 ng of pHIV-Pro-Luc or pHIV-ΔNF-κB-Pro-Luc were co-transfected into HeLa cells. After 6 h of transfection, the cells were treated with TA or Bryostatin-1 at different concentrations. Dual-luciferase reporter assay was performed at 48 h post transfection using the Promega Dual-Luciferase Reporter Assay System according to the manufacturer’s instructions.

### Genetic diversity analysis of activated HIV-1 viruses

The genetic diversity among HIV-1 quasispecies following various reagent treatments was evaluated through the sequencing of the env V1-V3 segment, employing the primer pairs previously reported (36). To mitigate potential sampling bias, a single genome amplification method was performed, and 50 independent PCR products obtained from each sample were used for cloning. The alignments of HIV-1 sequences were built by using MUSCLE, and all ambiguous positions were removed for each sequence pair. Subsequently, the average genetic distance between each clone and the relevant entire population in each sample was calculated using MEGA 6. Furthermore, the two-tailed Mann-Whitney *U*-test was performed using Graphpad Prism 8 software for comparing the genetic diversities between different samples. To further depict the globe landscape of HIV diversity, the phylogenetic bootstrap consensus trees were also constructed using neighbor-joining method with 1,000 bootstrap replications implemented in MEGA 6.

### Quantitative analysis of synergy of latency-reversing agent combinations

The Bliss independence model was used to predict the combined effects of TA with other LRAs (46). The Bliss independence model is defined by the equation fa_*xyP*_ = fa_*x*_ + fa_*y*_ – (fa_*x*_)(fa_*y*_), where fa_*xyP*_ is the predicted fraction affected by the combination of drug *x* and drug *y* given the experimentally observed fraction affected for drug *x* (fa_*x*_) and drug y (fa_*y*_) individually. Also, f_*xyO*_ is the observed fraction affected by a combination of drug *x* and drug *y*. With this model, Δfa_*xy*_ = *f*_*xyO*_ – fa_xy*P*_, if Δfa_*xy*_ > 0, the two drugs provide an indication of synergy. If Δfa_*xy*_ = 0, the drug combination follows the Bliss model for independent action, and if Δfa_*xy*_ < 0, the combined effect of the two drugs displays antagonism. The calculation of fa_*x*_ = (HIV RNA copies with drug *x*-background copies with DMSO)/(HIV RNA copies with PMA-background copies with DMSO). In our studies, J-Lat 10.6 cells were treated with TA, JQ-1, bryostatin-1, panobinostat, and SAHA individually, using DMSO as a negative control. At the same time, we conducted combination treatments of TA with JQ-1, bryostatin-1, panobinostat, and SAHA and compared the levels of activation to those of the individual treatment groups. The calculation of fa_x_ for J-Lat 10.6 cells used the percentage of GFP-positive cells in place of intracellular HIV RNA. The quantification of GFP-positive expression was ascertained utilizing flow cytometry, with gating on viable cells and assessing the expression levels of GFP within this subset of live cells through the fluorescein isothiocyanate (FITC) channel.

### Chromatin immunoprecipitation

ChIP assays were performed according to the manufacturer’s instructions (CST). In brief, four million cells in each group were crosslinked with 1% formaldehyde (Sigma-Aldrich) and lysed with Buffer A (CST) supplemented with dithiothreitol (DTT) and PIC. The nuclei were pelleted and digested with micrococcal nuclease (CST) in Buffer B (CST), followed by sonication with three sets of 20-second pulses at 40% amplitude. The supernatant, which contained digested chromatin, was clarified by centrifuging. One-tenth of the supernatant was proceeded to DNA purification to determine the size distribution and concentration of digested DNA. For each IP reaction, approximately 10 µg of chromatin was diluted into 500 µL of ChIP Buffer supplemented with PIC. Ten microliters of diluted chromatin was used as input control. ChIP antibodies against normal rabbit IgG (CST, 2729), H3K27me3 (Abcam, ab6002), or H3K9me3 (Abcam, ab8898) were added into each reaction and incubated for at least 6 h at 4°C while rotating. Antibodies-bound proteins and DNAs were pulled down with ChIP-Grade Protein G Magnetic Beads (CST) and eluted with ChIP Elution Buffer (CST). These enriched complexes and input samples proceeded with DNA purification. Purified DNA fragments in each group were quantitated by qRT-PCR, with HIV-1 LTR primers.

### Statistical analysis

Results of the experiments were presented as mean ± S.D. (error bars). *t*-test was used for the comparison of two sample means, and one-way analysis of variance (ANOVA) was used for the comparison of multiple groups’ means. Statistical analyses were conducted with Graphpad Prism 8. A value of *P* < 0.05 was considered to be statistically significant and represented as *, a value of *P* < 0.01 was considered to be more statistically significant and represented as **, and a value of *P*  <  0.001 was considered to be the most statistically significant and represented as ***.

## Data Availability

The data that support the findings of this study are available from the corresponding author upon reasonable request.

## SUPPLEMENTAL MATERIAL

Supplemental figures jvi.01173-24-s0001.pdf Figures S1 to S7.

ASM does not own the copyrights to Supplemental Material that may be linked to, or accessed through, an article. The authors have granted ASM a non-exclusive, world-wide license to publish the Supplemental Material files. Please contact the corresponding author directly for reuse.
