# Supplementary material for: Tannic acid reactivates HIV-1 latency by mediating CBX4 degradation
Source: J Virol. 2024 Dec 18;99(1):e01173-24. doi: 10.1128/jvi.01173-24 (PMC11790007; doi:10.1128/jvi.01173-24)
Supplement: Supplemental figures — Figures S1 to S7. [file jvi.01173-24-s0001.pdf]

Figure S1. Construction of Tat-sensitive HIV-1 latently-infected cell line model

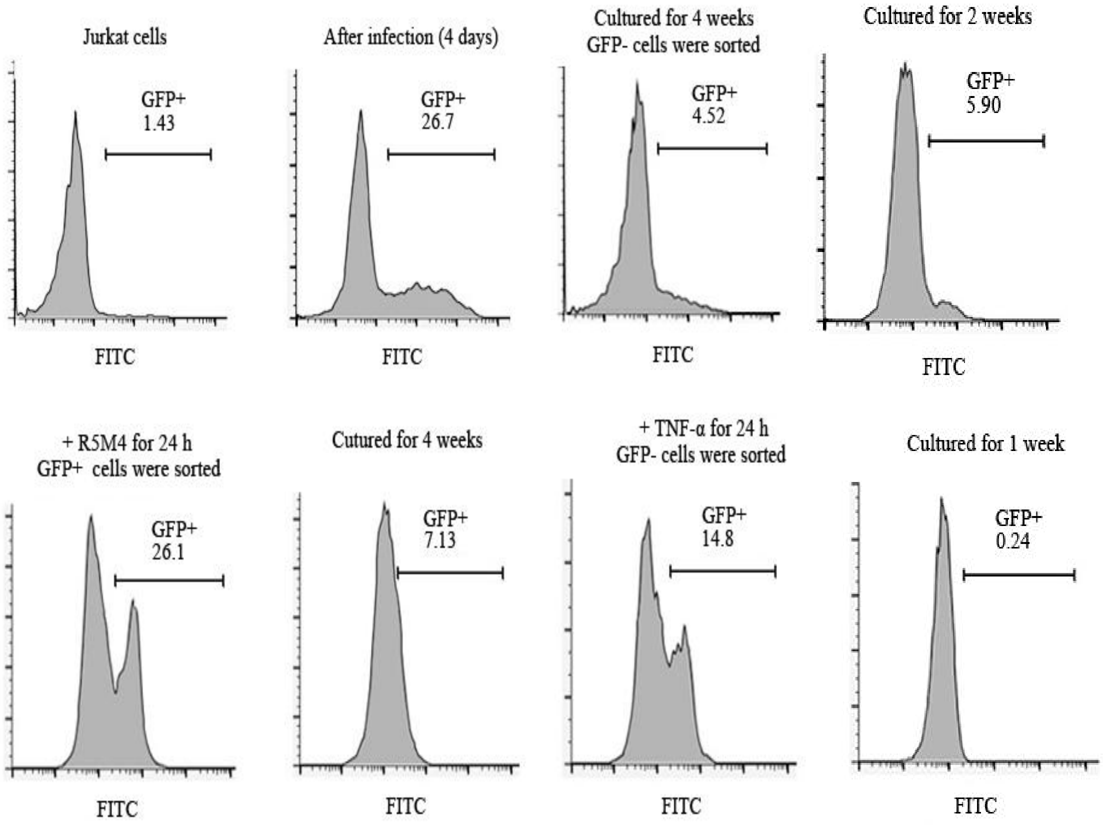

Figure S1. Construction of Tat-sensitive HIV-1 latently infected cell line model
The establishment process of Tat-sensitive HIV-1 latently infected cell line model, showing the proportion of GFP-positive cells at each stage.

Figure S2. Screening of Tat-like HIV-1 reservoir reactivators

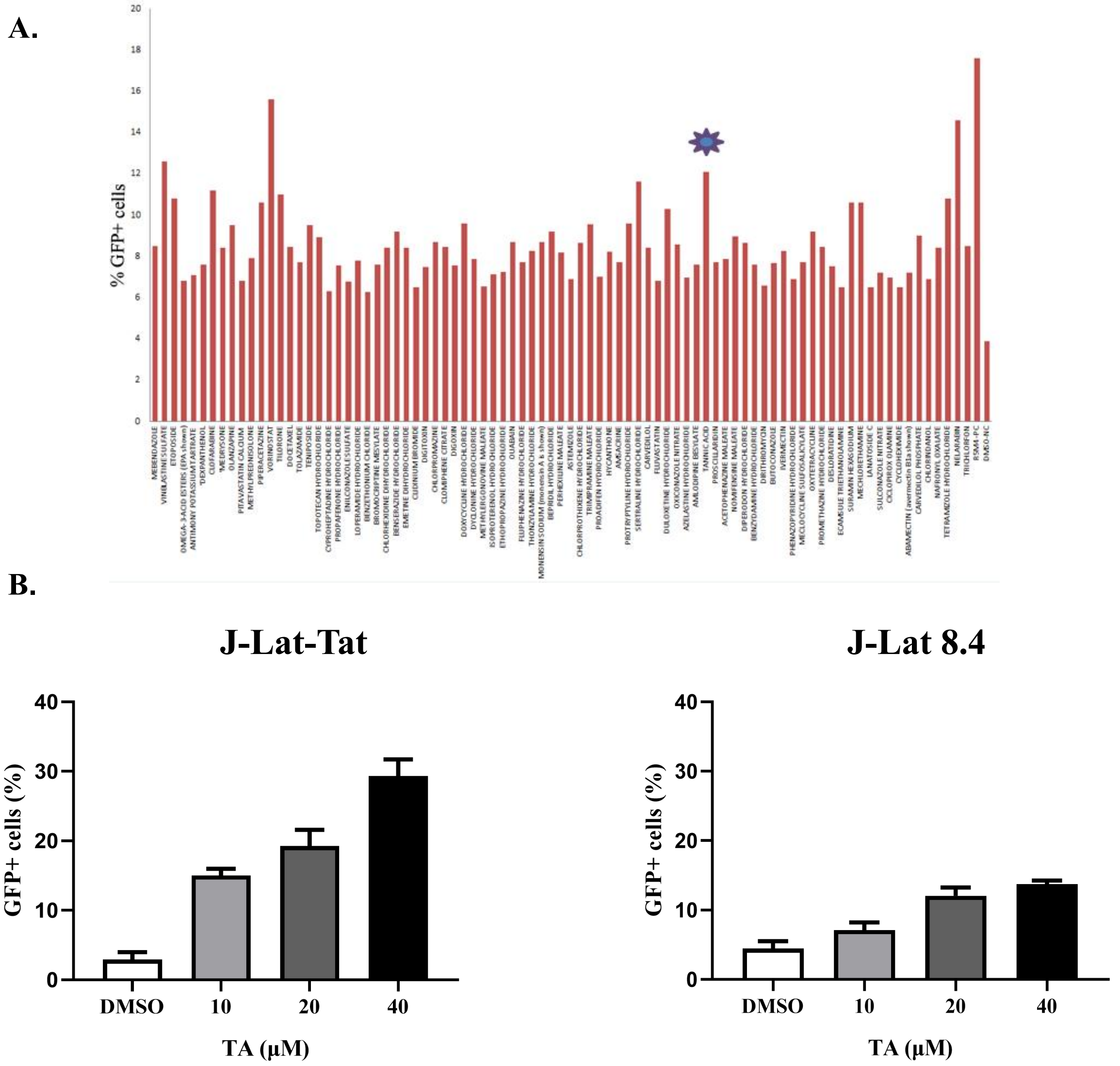

Figure S2. Screening of Tat-like HIV-1 reservoir reactivators

(**A**) Results of the first round of high-throughput screening from three independent experiments. The high-throughput screening process of the Tat-like HIV-1 reservoir reactivators by US Drug Collection. Cells were plated into 96-well plates, the drugs from US Drug Collection were added for 24 h. and the GFP expression was detected by high throughput flow cytometry. DMSO as a negative control, TNF-α, SAHA and R5M4 as positive control. (**B**) Reactivation efficiency of different concentrations of TA in two HIV-1 latently infected cell models, J-Lat-Tat and J-Lat 8.4.

Figure S3. The reactivation effect of TA in HIV-1 latently infected primary CD4^{+} T lymphocytes

A.

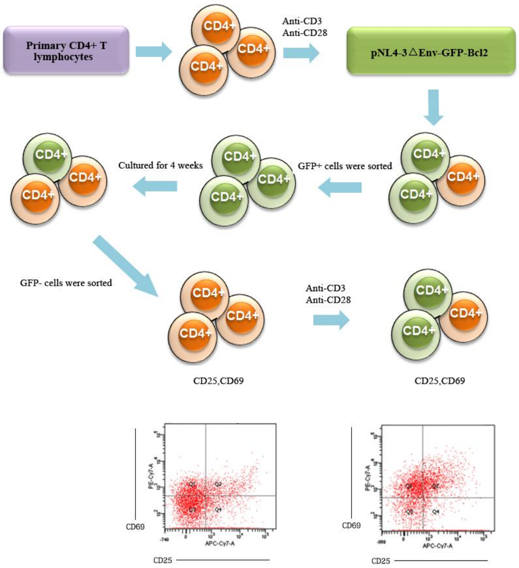

B.

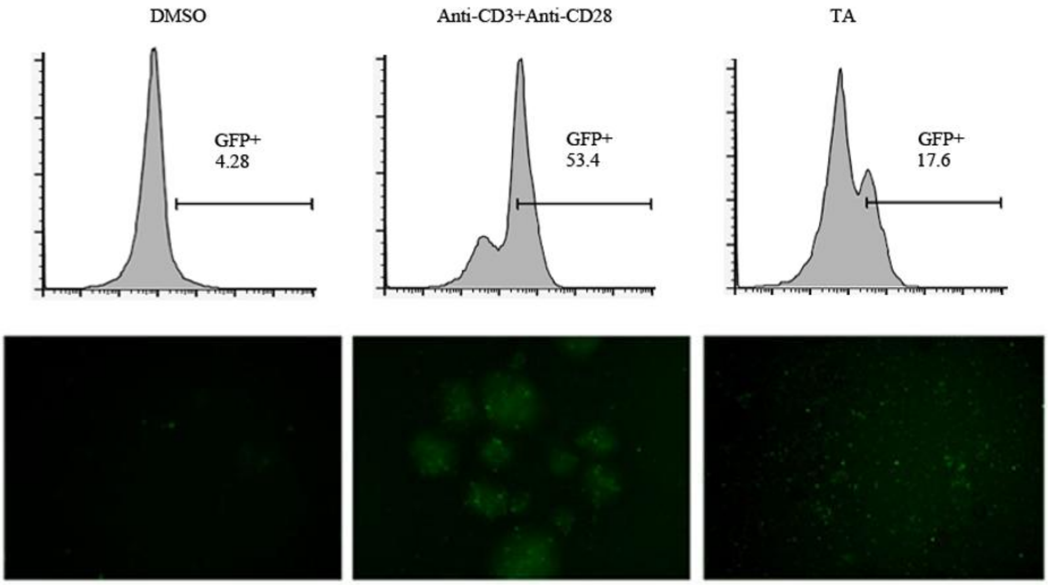

Figure S3. The reactivation effect of TA in HIV-1 latently infected primary CD4^{+} T lymphocytes
(**A**) Construction process of HIV-1 latently infected primary CD4^{+} T lymphocytes. PBMCs were isolated from healthy human peripheral blood and CD4^{+} T cells were sorted out with CD4^{+} magnetic beads according to the manipulation and cultured for 72 h. Then CD4+ T cells were stimulated with anti-CD3, anti-CD28 and IL-2, and the activated CD4^{+} T cells were infected with the pseudoviruses HIV-1$_{NL4-3}$-ΔEnv-GFP-Bcl2 for 6 h. After 4 days, the infection efficiency was detected, and GFP-positive cells were sorted by flow cytometry. The cells were cultured for 4 weeks to the resting state, and GFP-negative cells were sorted. To test the effect of these cells, anti-CD3, anti-CD28 were added and the expression of CD25 and CD69 were detected by flow cytometry. (**B**) The reactivation effect of TA on HIV-1 latently infected primary CD4^{+} T lymphocytes from flow cytometry and fluorescence microscopy.

Figure S4. The combination of TA with other LRAs in PWH

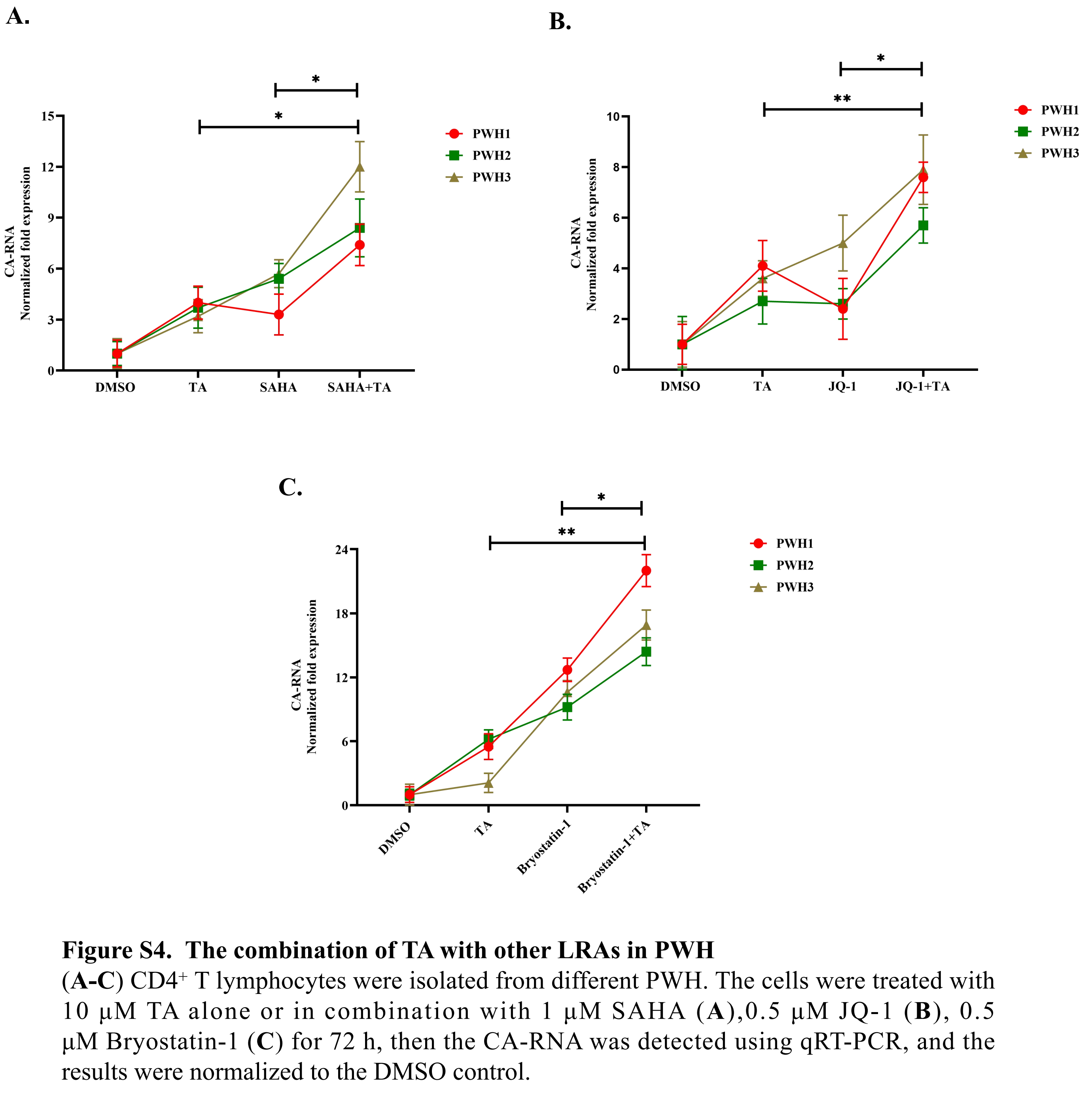

Figure S4. The combination of TA with other LRAs in PWH
(**A-C**) CD4+ T lymphocytes were isolated from different PWH. The cells were treated with 10 μM TA alone or in combination with 1 μM SAHA (**A**),0.5 μM JQ-1 (**B**), 0.5 μM Bryostatin-1 (**C**) for 72 h, then the CA-RNA was detected using qRT-PCR, and the results were normalized to the DMSO control.

Figure S5. TA reactivates HIV-1 LTR by down-regulating CBX4

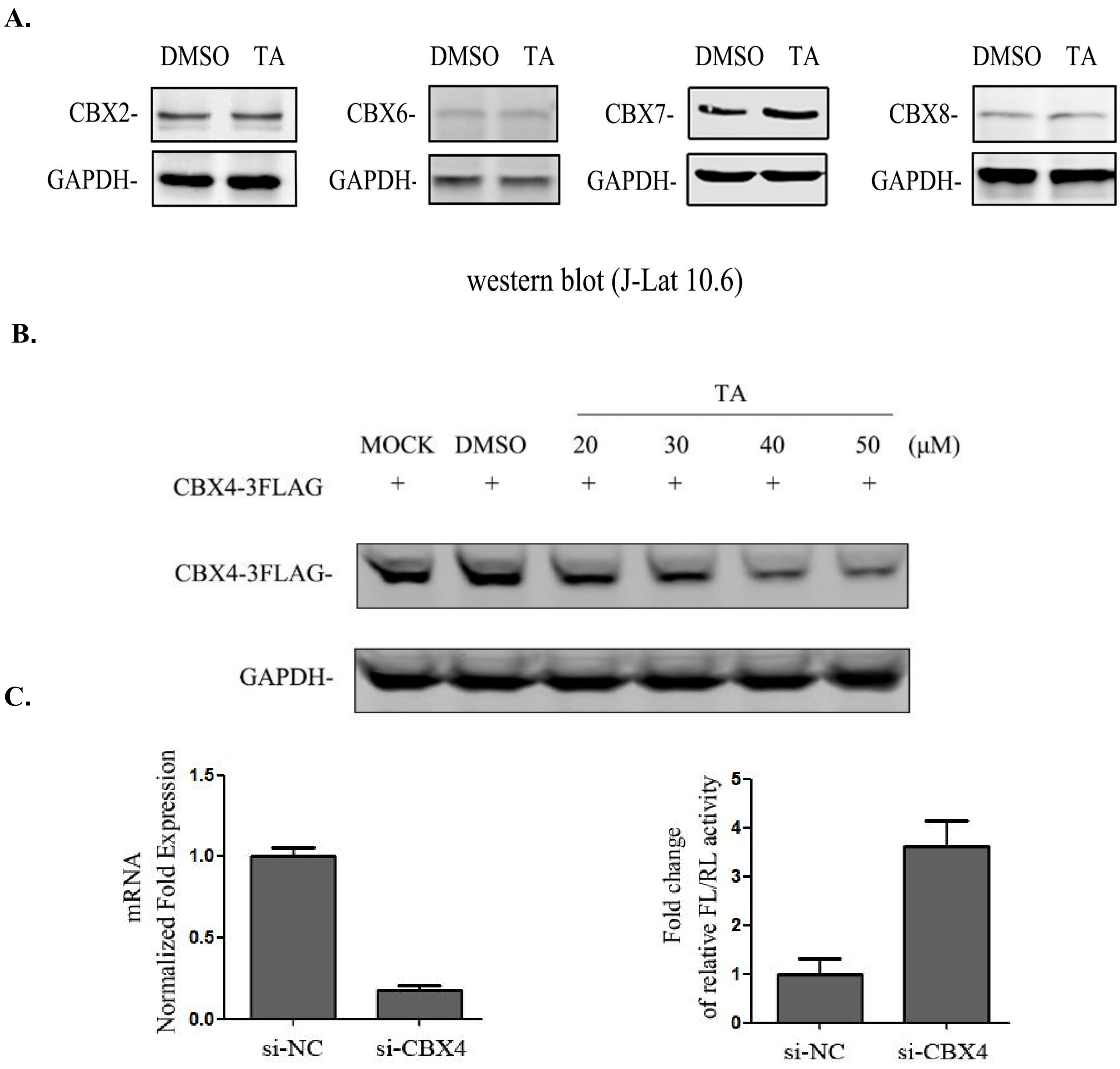

Figure S5. TA reactivates HIV-1 LTR by down-regulating CBX4
(**A**) Effect of TA on the expression levels of CBX2, CBX6, CBX7, CBX8 proteins. J-Lat 10.6 cells were treated with DMSO or TA for 24 h, and the corresponding endogenous proteins were detected by western blotting with anti-CBX2, anti-CBX6, anti-CBX7, anti-CBX8. (**B**) 2 μg pcDNA3.1-CBX4-3FLAG plasmid wass transfected into HeLa cells for 6 h, then the cells were treated with 20-50 μM TA, the expression of CBX4 was confirmed after 24-hour treatment by western blotting with anti-FLAG antibody. (**C**) HeLa cells were cultured in 12-well plates, siRNA-NC and siRNA-CBX4 were transfected into cells respectively. After 48 h, the knockdown efficiency of siRNA-CBX4 was tested. (**D**) Knockdown of CBX4 was able to activate HIV-1 LTR transcription effectively. TZM-bl cells were cultured in 24-well plates and transfected with siRNA-NC and siRNA-CBX4, respectively. 48 hours later, dual-luciferase assay was performed for LTR transcriptional activity.

Figure S6. TA downregulates the expression of CBX4 by E3 ligase CUL4A

A.

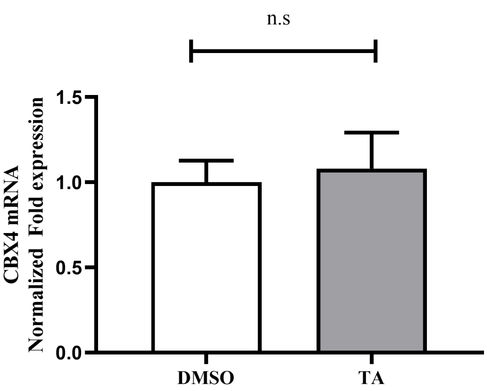

B.

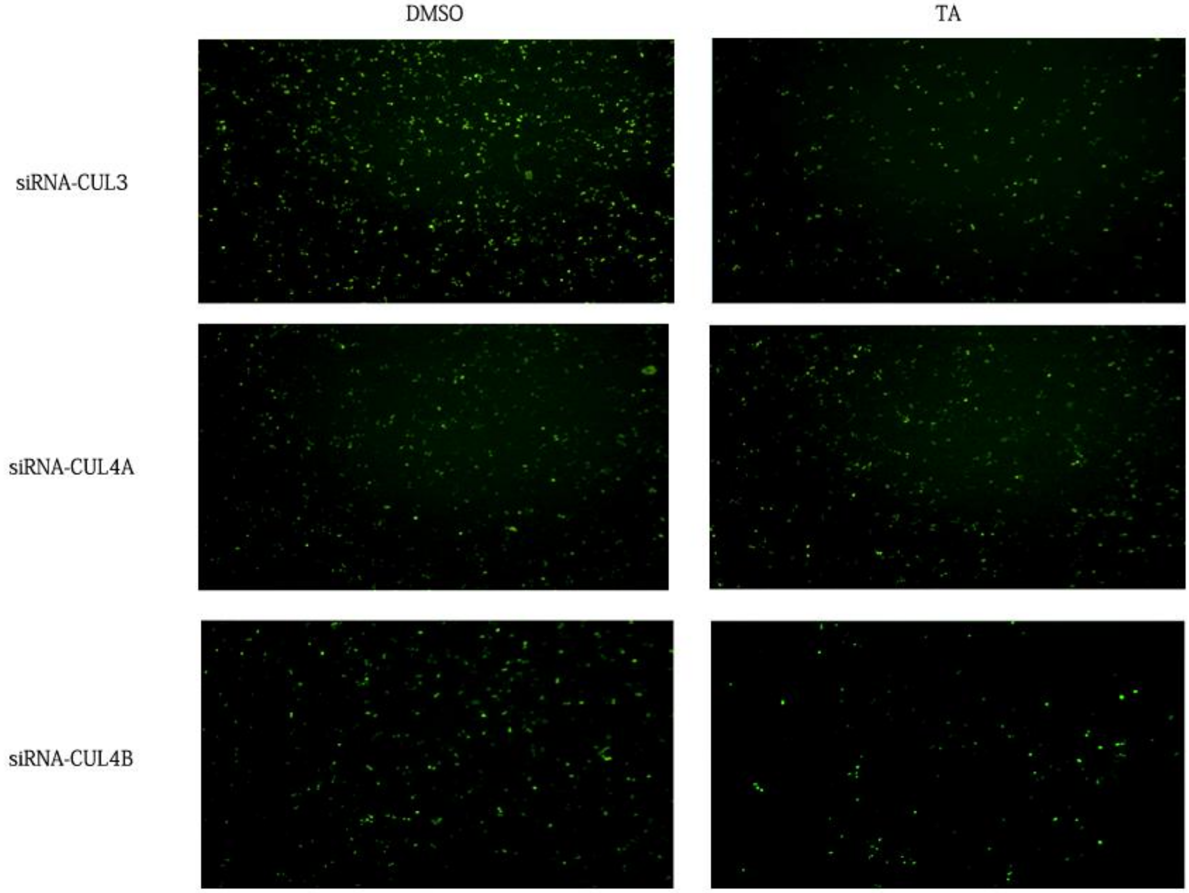

Figure S6. TA downregulates the expression of CBX4 by E3 ligase CUL4A

(**A**) TA had no significant effect on the expression of CBX4 mRNA. J-Lat 10.6 cells were treated with DMSO or TA for 24 h, mRNA levels of CBX4 were detected with qRT-PCR. (**B**) CUL4A is a key factor in the degradation of CBX4 by TA. HeLa cells were cultured in 6-well plates, siRNA-NC, siRNA-CUL3, siRNA-CUL4A, siRNA-CUL4B and pEGFP-CBX4 were cotransfected into the cells, respectively. After 6 h, DMSO or TA treatment was added for 24 h, then the fluorescence expression of pEGFP-CBX4 was detected.

Figure S7. TA downregulates the expression of CBX4 via increasing the ubiquitination of CBX4 in HeLa cells

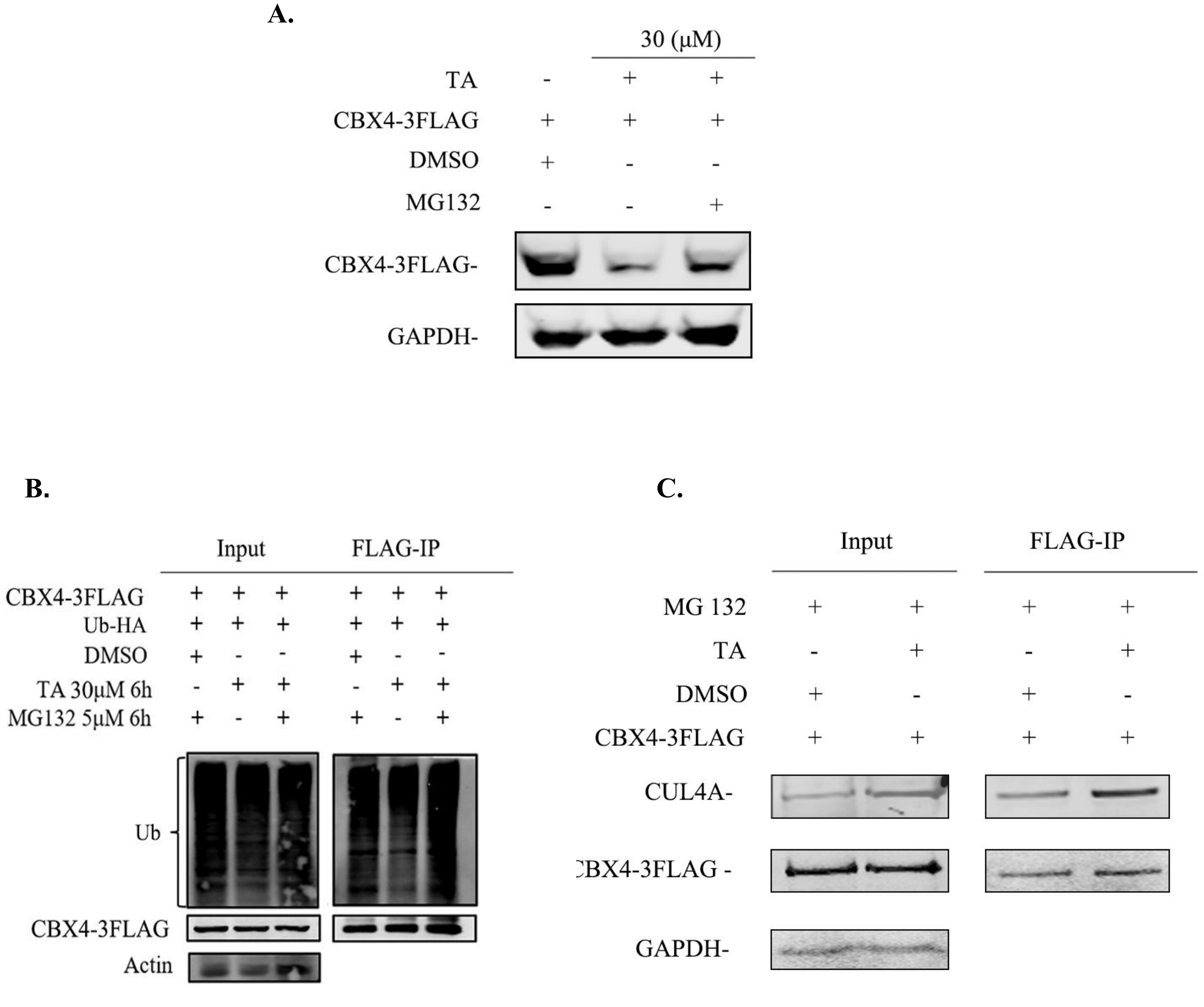

Figure S7. TA downregulates the expression of CBX4 via increasing the ubiquitination of CBX4 in HeLa cells
(A) HeLa cells were transfected with 2 μg pcDNA3.1-CBX4-3FLAG, after 6 h, 30 μM TA and 4 μM MG132 were added for 16 h and detected with western blotting. **(B)** 4 μg pcDNA3.1-CBX4-3FLAG and 4 μg pcDNA3.1-Ub-HA were transfected into HeLa cells. After 6 h cells were treated with 30 μM TA and 4 μM MG132 for 16 h, and analyzed by co-immunoprecipitation with anti-FLAG agarose beads, and analyzed by western blotting assay with anti-FLAG, anti-HA. (**C**) HeLa cells were transfected with pcDNA 3.1-CBX4-3FLAG, then treated with 30 μM TA and 4 μM MG132 for 16 h. Samples were immunoprecipitated with anti-FLAG agarose beads and analyzed by western blotting with anti-CUL4A, anti-FLAG antibodies.
